# Whole-Body Cryostimulation - Potential Beneficial Treatment for Improving Antioxidant Capacity in Healthy Men - Significance of the Number of Sessions

**DOI:** 10.1371/journal.pone.0046352

**Published:** 2012-10-15

**Authors:** Anna Lubkowska, Barbara Dołęgowska, Zbigniew Szyguła

**Affiliations:** 1 Faculty of Health Sciences, Pomeranian Medical University, Szczecin, Poland; 2 Department of Physiology, Faculty of Biology, Szczecin University, Szczecin, Poland; 3 Department of Laboratory Diagnostics and Molecular Medicine Pomeranian Medical University, Szczecin, Poland; 4 Institute of Human Physiology, University School of Physical Education, Krakow, Poland; Institut Pluridisciplinaire Hubert Curien, France

## Abstract

It is claimed that WBC (whole-body cryotherapy) enhances the resistance of the human body, also thanks to the beneficial effect on the antioxidant system. Accordingly, this research aimed to evaluate the effect of a series of whole-body cryostimulations on the level of non-enzymatic antioxidants and the activity of antioxidant enzymes in healthy men. The study was carried out on 30 young and healthy men aged 27.8±6.1 years with average body mass index and peak oxygen consumption (46.34±6.15 ml kg^−1^ •min^−1^). The participants were daily exposed for 3 minutes to cryogenic temperatures (−130°C). Blood samples were obtained in the morning before cryostimulation, again 30 min after exposure and the following day in the morning, during the 1^st^, 10^th^ and 20^th^ session. Analysis concerned changes in plasma concentrations of total protein, albumin, glucose, uric acid and ceruloplasmin, and the most important components of the antioxidant system in red blood cells: superoxide dismutase, catalase, glutathione peroxidase, glutathione reductase, reduced and oxidized glutathione. To assess the oxidative stress level the 8-isoprostane concentration in plasma was measured. The obtained results indicate that cryogenic temperatures in repeated daily treatments result in changes in the peroxidant and antioxidant status. These changes seem to depend on the number of cryostimulations. After 20 daily treatments there was an increase in SOD, SOD:CAT ratio, a decrease in the concentration of reduced and oxidized glutathione and in the activity of GPx. It could be possible that differences in the activity of GSSG-R after 20 treatments depended on the body mass index of participants.

## Introduction

Short-term exposure to cryogenic temperatures applied locally or over the whole body is increasingly used to obtain stimulation of the body via physiological reactions to cold. Although efferent pathways to thermoeffectors in humans have not been fully determined, it has been observed that the physiological and biochemical thermoregulatory reactions to cold involve the vascular system, skeletal muscle and brown adipose tissue (BAT) [1]. Sudden cold exposure also stimulates intense sympatho-adrenal activity, eliciting secretion of norepinephrine, epinephryne and cortisol [2], [3]. Exposure to cold results in alternating periods of constriction and diastole of vascular vessels, known as ‘hunting reaction’ and ‘rebound effect’ especially in fingers and toes [4], which may lead to reperfusion and related phenomena. The ability to maintain homeothermia requires the initiation of shivering thermogenesis in muscles and non-shivering thermogenesis in the adipose tissue. Increased heat production during cold exposure through changes in metabolism and shivering thermogenesis can even induce an increase in body temperature during cold exposure (Tb) since 70–100% of the metabolic energy released for muscular work is converted to heat [5]. We hypothesized that aforementioned changes during whole body cryostimulation (WBC) may lead to an increase in free radical reactions in the body and the production of reactive oxygen species (ROS), which may be associated by compensatory adaptation changes. It is postulated that cryostimulation stimulates antioxidant responses and enhances favorable adaptation mechanisms [6] but research on the influence of cryostimulation on the production of reactive oxygen species (ROS), lipid peroxidation and the antioxidant response of the body, has focused mostly on the treatment of people with different diseases [7]. It is assumed that the effect of those reactions may be beneficial for human health, especially in repeated controlled exposure to whole-body cryostimulation (WBC), but the mechanism and dose dependence is still unknown. Some authors discuss the role of cryostimulation in sport, its effectiveness in athletic renewal and protective activity against the impairment of muscle fibers during effort [5], [8], [9], [10]. However, there are not too many studies on the effect of WBC on the prooxidant and antioxidant status in clinically healthy individuals exposed to cryostimulation without clinical indications. In our previous studies we showed that both single WBC and a series of 10 daily cryostimulations caused changes in the plasma peroxidant-antioxidant balance in healthy men [11], [12], [13]. Importantly, we have also observed that the beneficial character of biochemical changes (e.g. in lipid levels, anti-inflammatory cytokine level) was visible only after a longer series of exposures (20 sessions) and for a series of 10 stimulations the changes were not so significant [14], [15]. From the therapeutic point of view it is interesting what is the real stress level in response to one and prolonged cryostimulation treatment and if the repeated stress factor, in this case extremely low temperatures, may lead to a compensatory increase in the activity of antioxidant enzymes and non-enzymatic antioxidants. Miller et al. [7], [16] suggested that 10 session of cryostimulation may reduce generation different reactive oxygen and nitrogen species (ROS/RNS) because lead to increase of superoxide dismutase (SOD), uric acid (UA) and total antioxidant status in plasma (TAS) in multiple sclerosis patients and healthy patient. Additionally those authors didn't observe the increase in thiobarbituric acid reactive substance (TBARS) as a marker of lipid peroxidation. But maybe the lack of association may be due to the TBARS have been criticized as being a nonspecific marker of lipid peroxidation [17], [18].

There is also a question of the optimal number of sessions. The routine number in a series is 10 daily exposures, although there are no unambiguous and scientifically proven indications for the use of a specific number of stimulations. Very often the use of a specific number of sessions in a series results from own experience and recommendations of cryochamber manufacturers, often without rational and scientific explanation. Therefore in this study we extended the series to 20 WBC sessions, this time performed on a larger group of participants than in our previous studies. Our analysis concerned changes in the most important components of the antioxidant system: superoxide dismutase (SOD, E.C.1.15.1.1), catalase (CAT, E.C.1.11.1.6), glutathione peroxidase (GPx, E.C. 1.11.1.9), glutathione reductase (R-GSSG, E.C.1.8.1.7), reduced (GSH) and oxidized (GSSG) glutathione, ceruloplasmin and uric acid. In order to assess the real level of stress induced by the repeated exposure to cryogenic temperatures over the whole series of WBC, we examined the concentration of 8-isoprostane (Iso-P) in plasma, which is one of the family of eicosanoids. Isoprostanes are considered the best available biomarkers of oxidative stress status and lipid peroxidation in vivo [19].

## Materials and Methods

### Subjects

The research was carried out on 30 men, aged 27.8±6.1 years, who had never been subjected to any form of cryostimulation. All the subjects were healthy and normotensive, with a body mass index (BMI) between 22.12 and 33.20. Among the 30 examined individuals, the BMI was below 25 in 17 individuals and in the remaining 13 the BMI was above 25. We decided to analyze if there were any BMI-dependent differences in the activity of antioxidant enzymes during exposure to cold.

All subjects were volunteers and were fully informed about the study protocol, of any risks and discomfort associated with the experimental procedure, and their rights according to the Declaration of Helsinki. All participants accepted to participate and completed the written informed consent and health history questionnaire. The study was approved by the local Ethics Committee (Etics Committee of Pomeranian Medical University; Ref.KB-0012/54/10).

Additionally before the experiment, each participant was examined by a physician in order to find any contraindications against cryostimulation in the given individual. Basic anthropometric data was collected, including body height, body mass and body mass index (BMI). Body mass index was calculated as weight (in kilograms) divided by height (in meters) squared. In the week before cryostimulation, the participants were subjected to tests of maximal physical performance, establishing their maximal oxygen uptake (VO_2_max) using a direct method described previously [20]. Arterial blood pressure [mmHg] was measured before the exercise on the brachial artery in a sitting position using a sphygmomanometer with an accuracy of 5 mmHg.

### Cryostimulation procedure

Participants were exposed to a 3 min session of extremely low temperature (−130°C) in a two-stage cryogenic chamber every day at the same time between 9 am and 10 am for 20 consecutive days. Entry to the cryochamber was preceded by an approximately 30-second period in the vestibule at a temperature of −60°C. Glasses, contact lenses and all jewellery were removed before entry to the chamber as well as drying the body thoroughly to eliminate the sensation of cold, noses and mouths were secured with a surgical mask. During the cryostimulation procedure, the subjects were dressed only in shorts, socks, wooden clogs, gloves and a hat covering the auricles against frostbite. While in the cryochamber the subjects were advised to animate fingers and legs slightly and to avoid holding the breath. The participants were obliged to maintain the same level of physical activity and regular diet for the period of the tests.

### Sample collection

Blood was collected during the 1st, 10th and 20th days of the whole-body cryostimulation series, in the following order: A – after overnight fasting, in the morning between 8.00 and 8.30 am, before the first cryostimulation; B - 30 minutes after the first cryostimulation; C – 24 hours after the first cryostimulation, after overnight fasting, between 8.00 and 8.30 am; D – in the morning before the 10^th^ cryostimulation, after overnight fasting; E - 30 mins after the 10^th^ cryostimulation; F – 24 hours after the10^th^ cryostimulation, after overnight fasting; G – in the morning before the 20^th^ cryostimulation, after overnight fasting; H - 30 mins after the 20^th^ cryostimulation; I – 24 hours after the 20^th^ cryostimulation, after overnight fasting.

Venous blood samples were collected from the antecubital vein 10 minutes after resting in a sitting position using vacutainer system tubes (Sarstedt, Germany), separately to two tubes: to determine blood variables (1.2 ml; anticoagulated with 1 g/L EDTA), number of erythrocytes (RBC), hemoglobin concentration (HGB), hematocrit value (HCT), number of leukocytes (WBC), number of thrombocytes (PLT), and for biochemical analysis of erythrocytes and plasma (7 ml; EDTA). Hematological parameters were measured by the use of an hematology analyzer (Micros 60). Separately for GSSG assay we used the thiol-scavenging reagent, 1-methyl-2-vinylpyridinium trifluoromethanesulfonate1 (M2VP) at a level that rapidly scavenges GSH but does not interfere with the GR assay (10 µL M2VP added to 100 µL whole blood). The erythrocytes were separated by centrifugation (300 rpm, 1500 G, 10 min, 4°C), washed three time with buffered NaCl solution (PBS: 0.01 mol phosphate buffer 0.14 mol NaCl, pH 7.4) chilled to 4°C and finally frozen at −70°C. Plasma was divided into aliquots and immediately deep-frozen at −70°C until the time of analysis, however not longer than 30 days.

### Biochemical measurements

Biochemical parameters such as: concentration of total protein, albumin, glucose and uric acid, were measured in the plasma samples using colorimetric methods on a diagnostic kit (BioMAxima, Poland). The ceruloplasmin concentration in plasma samples was determined by immunoturbidimetric method using a diagnostic kit (Cormay, Poland). ELISA kits were used for measuring serum levels of 8-isoprostane (Cayman, MI, USA), according to the manufacturer's protocol. Each sample was tested in duplicate.

### Analysis of erythrocyte anti-oxidative enzyme

Before the analysis, erythrocytes were thawed and the haemolysate of the washed red blood cells was diluted with distilled water and chilled to 4°C.

GSH and GSSG concentration in hemolysate samples was determined by the colorimetric method (Bioxytech GSH/GSSG-412 Kit, OxisResearch). SOD, CAT, GPx and R-GSS activity were also measured with a BIOXYTECH® kit (Oxis Research, Portland, OR, USA) using a UV/VIS Lambda 40 (Perkin-Elmer, Wellesley, MA, USA) spectrophotometer. SOD: sensitivity: 0.1 U/mL; specificity: 97%, coefficient of variation: lower than 4%; CAT: sensitivity: 1.71 U/mL, specificity: 89%, coefficient of variation: lower than 2%; GPx: sensitivity: 6 U/L, specificity: 94%, coefficient of variation: lower than 4%, GSSG-R: sensivity:0.14 U/L, specificity: 94%, coefficient of variation: lower than 4%; GSH/GSSG: sensitivity: 5 µmol/L, specificity: 95%, coefficient of variation: lower than 2%. The enzyme activity and glutathione concentration was calculated per 1 g of erythrocyte haemoglobin. In all mentioned cases haemoglobin levels were assayed using the Drabkin method.

### Statistical Analyses

The obtained results were statistically analysed. Distributions were examined using a Shapiro-Wilk test that indicated that some variables deviated from a normal distribution (were lognormal). Each studied parameter was characterised by: sample size, arithmetic mean/median, standard deviation. Data were tested by one-way ANOVA. Since in some cases the distribution was not normal, when a significant F-value was found, a Friedman post hoc test was performed and a nonparametric Wilcoxson post-hoc test for a dependent variable was performed. The accepted level of significance was defined as P<0.05. Development of statistical results was performed using STATISTICA PL v 7.1 software (Statsoft, Krakow, Poland). Significance was assumed at p<0.05.

## Results

Table 1 presents the basic anthropometrical and physiological parameters of the examined group. Based on the assessment of the oxygen threshold, we found that the respondents showed good physical performance (VO _2max_ = 46.34±6.15 ^mL.^ Kg^−1^ Min^−1^). In the case of all participants, the values of all hematological indices were within clinical and laboratory reference values. Table 2 shows changes in biochemical indices. Only the first day of treatment showed a marked decrease in the concentration of total protein, 30 minutes after leaving the cryogenic chamber (p≤0.05) - on the morning of the next day concentrations of proteins were similar to baseline. After 10 sessions there was a distinct increase in total protein concentration, maintained with continued exposure (by 13% after 20 sessions). Albumin concentration did not change but after 20 sessions, plasma level decreased by 12%. There were no changes in glucose - only the first session resulted in a significant but temporary increase in blood glucose levels, as further exposure did not cause any changes in blood glucose. 10 sessions resulted in a reduction of uric acid concentration, but 20 sessions resulted in a significant increase, 7% above the level prior to cryostimulation. Similarly, in the case of ceruloplasmin, 10 sessions did not cause any change in the concentration and only after 20 sessions was there a statistically significant 26% reduction in concentration.

**Table 1:** Anthropometrical and physiological characteristics of the study group.

	Means ± (SD)	Min	Max
Body height [cm]	177.4±6.9	163.0	184.0
Body mass [kg]	86.5±10.5	66.0	92.4
BMI [kg/m^2^]	26.8±3.7	22.1	33.2
SBP [mmHg]	124±6	106	128
DBP [mmHg]	73±8	65	80
HR _rest_ (bpm)	70±8	63	82
HR _max_ (bpm)	195.7±7.4	192	201
VO_2max_ [mL^.^kg^−1.^min^−1^]	46.3±6.1	44.6	53.1
WBC [10∧3/µL]	6.0±1.3	4.7	9.4
RBC [10∧6/µL]	4.8±0.3	4.0	5.4
HGB [g/dL]	15.2±1.0	12.5	17.0
HCT [%]	43.8±3.2	36.10	48.3
PLT [10∧3/µL]	238.9±45.9	153.0	323.0

Legend Table 1: BMI - body mass Index; VO_2max_ - maximal oxygen uptake, SBP - systolic blood pressure; DBP - diastolic blood pressure; HR_rest_ - heart rate; HR_max_ - post exercise heart rate; WBC- White body cell; RBC - Red body cell; HGB - Hemoglobin; HCT - Hematocrit; PLT - platelets; SD - standard deviation. Values are expressed as means ± SD, Minimum and Maximum.

**Table 2:** The plasma concentration of the 8-isoprostane, non-enzymatic components and some of the biochemical parameters before(A) and in response to a one (B,C), 10 (D,E,F)and 20 (G,H,I) sessions of whole-body cryostimulation.

		8-Isoprostane [pg/mL]	Total protein (g/dL)	Albumin (g/dL)	Glucose (mg/dL)	Uric acid (mg/dL)	Ceruloplasmin (g/L)
1^st^ session	A	148.78±31.8	6.94±0.80	4.82±0.74	85.64±10.19	5.98±1.43	0.26±0.01
	B	141.99±30.31	6.27±1.37* ^A^	4.79±0.8	90.82±14.55* ^A^	6.04±0.48	0.25±0.03
	C	175.93±48.25* ^A^	6.92±1.22	4.74±0.87	82.05±17.7	5.75±1.65	0.25±0.04
10^th^ session	D	308.39±55.85*** ^A^	7.6±1.72* ^A^	4.85±0.80	82.85±17.87	5.32±1.8* ^A^	0.23±0.03
	E	323.93±49.89	7.56±1.76* ^A^	4.72±0.74	84.86±11.94	5.33±1.41* ^A^	0.25±0.01
	F	322.39±47.17	7.73±1.65* ^A^	4.59±0.60	86.03±12.2	5.40±1.33* ^A^	0.26±0.05
20^th^ session	G	312.56±46.74*** ^A^	7.64±0.95* ^A^	4.45±0.81* ^A^	82.47±14.2	6.67±1.07* ^A^	0.18±0.05* ^A,F^
	H	308.13±39.02	7.59±0.78* ^A^	4.33±0.65* ^A^	84.69±16.2	6.68±1.41* ^A^	0.18±0.03* ^A,D,F^
	I	285.73±19.31* ^G^	7.89±1.04* ^A^	4.21±0.57* ^A^	82.90±14.36	6.42±1.25* ^A^	0.19±0.05* ^A,F^

Legend Table 2:

* p≤0.05 statistically significant difference,

** p≤0.01 statistically significant,

*** p≤0.001; A – after overnight fasting, in the morning before the first cryostimulation; B - 30 min after the first cryostimulation; C - the next morning after the first cryostimulation, after overnight fasting; D – after overnight fasting, in the morning before the 10^th^ cryostimulation ; E - 30 min after the10^th^ cryostimulation; F - the next morning after the10^th^ cryostimulation, after overnight fasting; G - after overnight fasting, in the morning before the last (20^th^) cryostimulation; H - 30 min after the 20^th^ cryostimulation; I - the next morning after the 20^th^ cryostimulation, after overnight fasting. Values are expressed as means ± SD.

**Figure 1:**
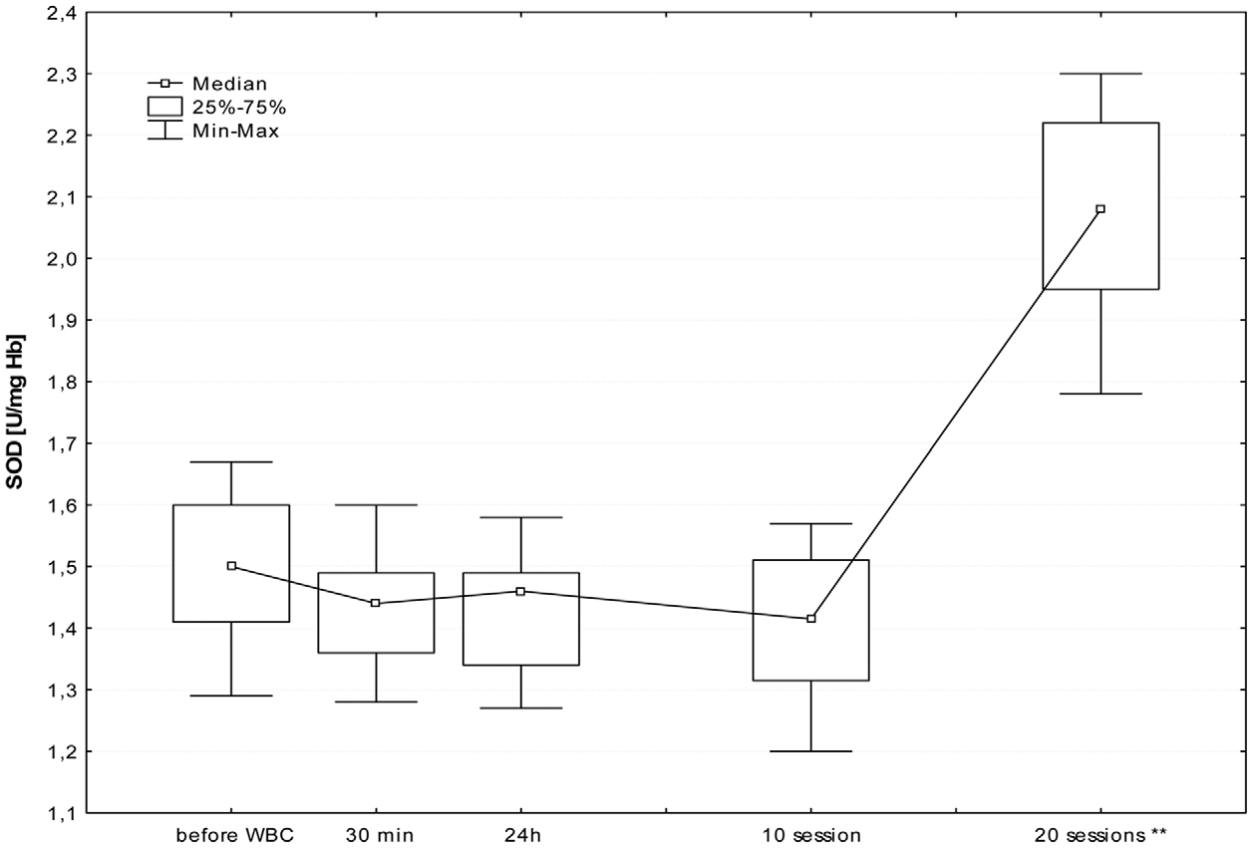
Changes in superoxide dismutase (SOD) activity in erythrocytes before and during following sessions of WBC. Legend Figure 1: Median and 25%–75% confidence interval; **p≤0.01 statistically significant vs. before WBC; before WBC - A: before the first cryostimulation, after overnight fasting; 30 min - B: 30 min after the first cryostimulation; 24 h – C: 24 hours after the first cryostimulation, after overnight fasting; 10 session – F: 24 hours after the10^th^ cryostimulation, after overnight fasting; 20 session – I: 24 hours after the 20^th^ cryostimulation, after overnight fasting.

**Figure 2:**
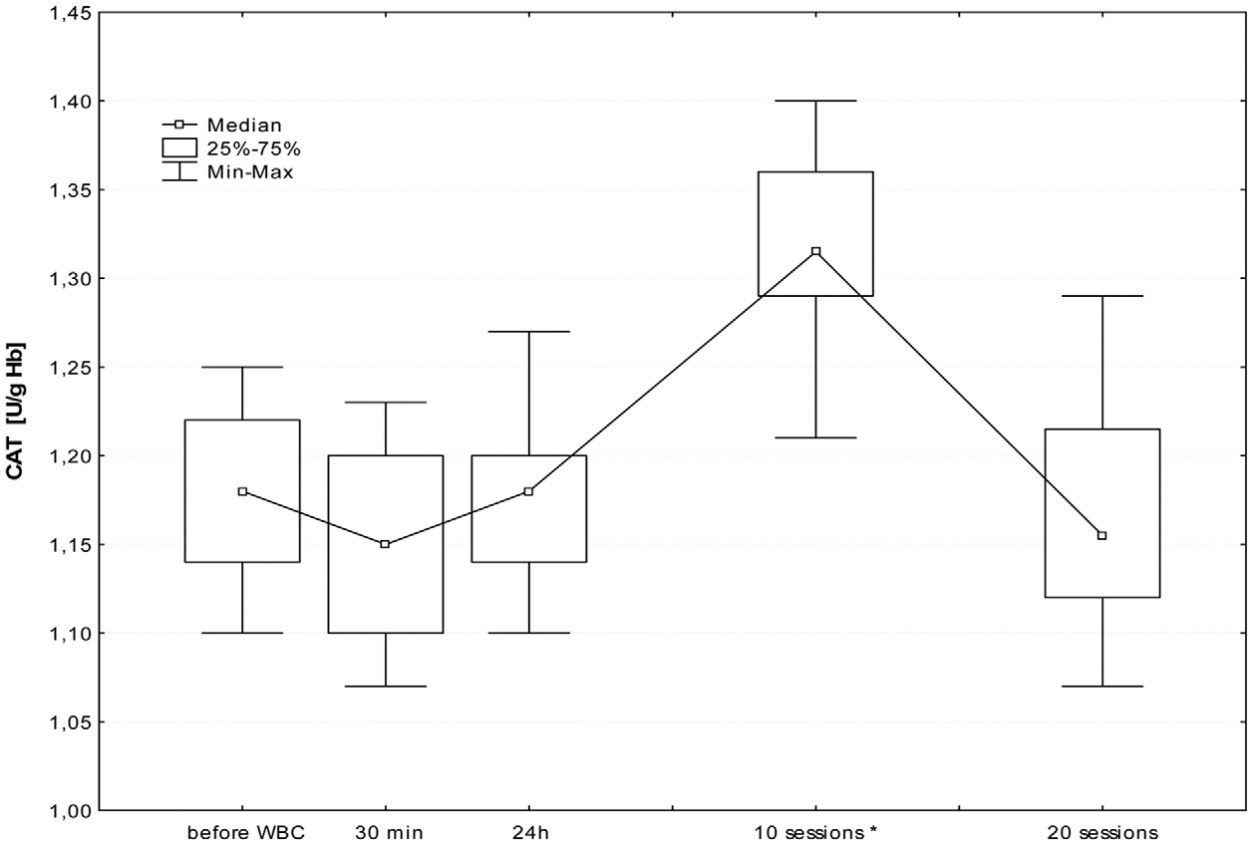
Changes in catalase (CAT) activity in erythrocytes before and during following sessions of WBC. Legend Figure 2: Median and 5%–95% confidence interval; * p≤0.05 statistically significant difference vs. before WBC; before WBC - A: before the first cryostimulation, after overnight fasting; 30 min - B: 30 min after the first cryostimulation; 24 h – C: 24 hours after the first cryostimulation, after overnight fasting; 10 session – F: 24 hours after the10^th^ cryostimulation, after overnight fasting; 20 session – I: 24 hours after the 20^th^ cryostimulation, after overnight fasting.

**Figure 3:**
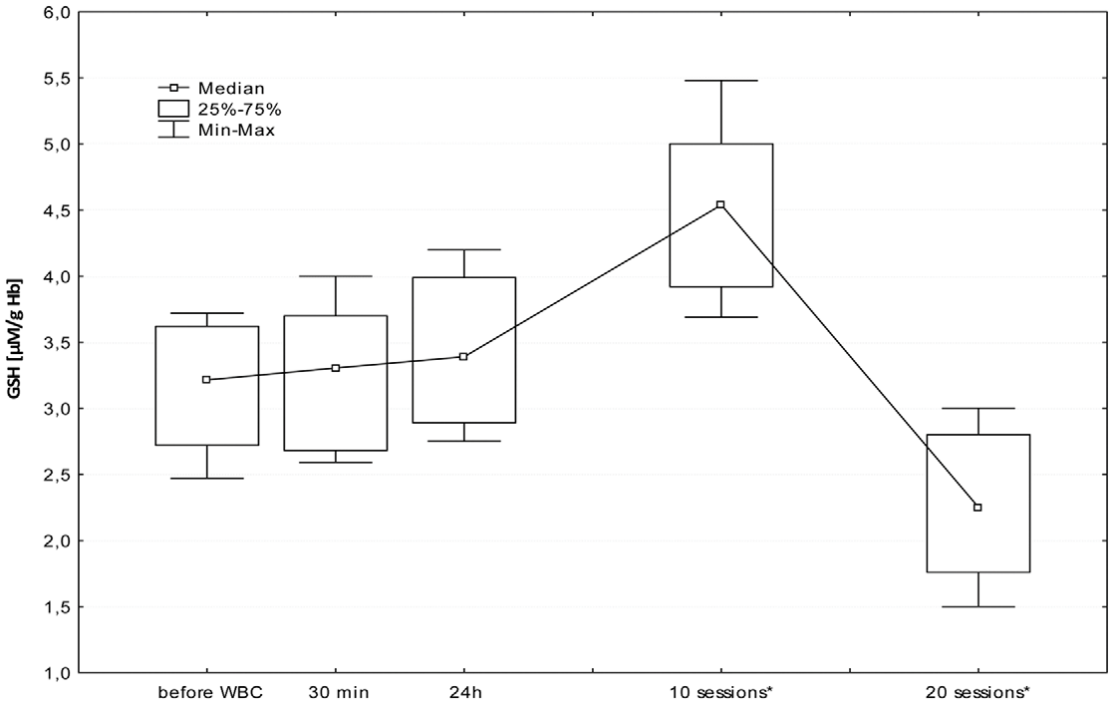
Changes in reduced glutathione (GSH) concentration in erythrocytes before and during following sessions of WBC. Legend Figure 3: Median and 5%–95% confidence interval; * p≤0.05 statistically significant difference vs. before WBC; before WBC - A: before the first cryostimulation, after overnight fasting; 30 min - B: 30 min after the first cryostimulation; 24 h – C: 24 hours after the first cryostimulation, after overnight fasting; 10 session – F: 24 hours after the10^th^ cryostimulation, after overnight fasting; 20 session – I: 24 hours after the 20^th^ cryostimulation, after overnight fasting.

**Figure 4:**
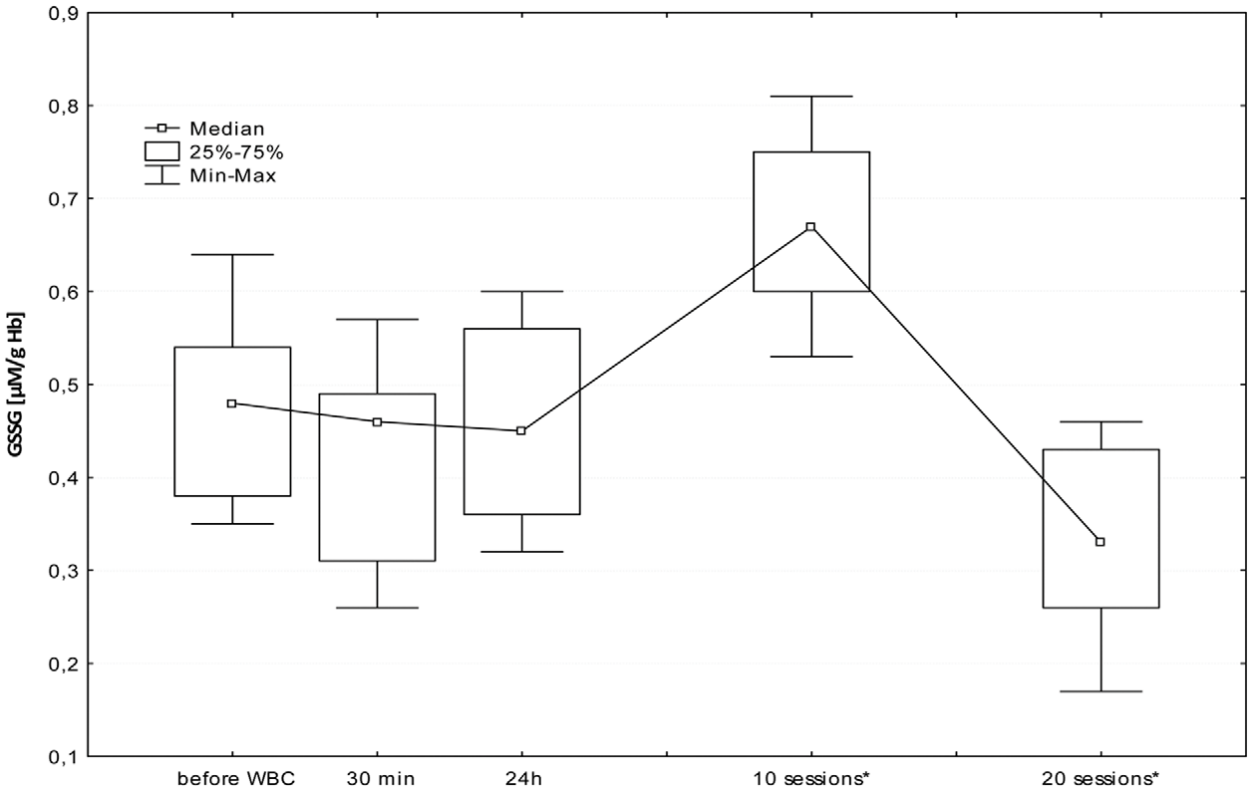
Changes in oxidized glutathione (GSSG) concentration in erythrocytes before and during following sessions of WBC. Legend Figure 4: Median and 5%–95% confidence interval; * p≤0.05 statistically significant difference vs. before WBC; before WBC - A: before the first cryostimulation, after overnight fasting; 30 min - B: 30 min after the first cryostimulation; 24 h – C: 24 hours after the first cryostimulation, after overnight fasting; 10 session – F: 24 hours after the10^th^ cryostimulation, after overnight fasting; 20 session – I: 24 hours after the 20^th^ cryostimulation, after overnight fasting.

**Figure 5:**
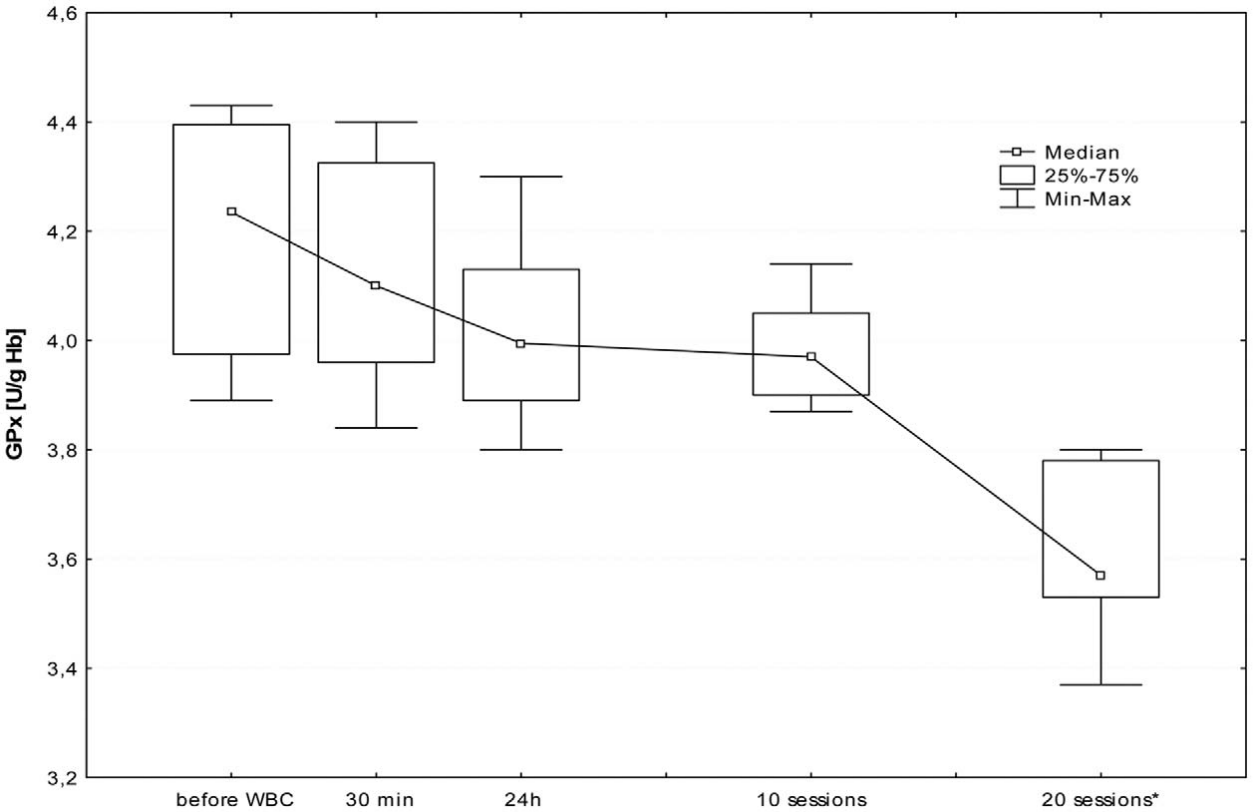
Changes in glutathione peroxidase (GPx) before and during following sessions of WBC. Legend Figure 5: Median and 5%–95%; * p≤0.05 statistically significant difference vs. before WBC; before WBC - A: before the first cryostimulation, after overnight fasting; 30 min - B: 30 min after the first cryostimulation; 24 h – C: 24 hours after the first cryostimulation, after overnight fasting; 10 session – F: 24 hours after the10^th^ cryostimulation, after overnight fasting; 20 session – I: 24 hours after the 20^th^ cryostimulation, after overnight fasting.

**Figure 6:**
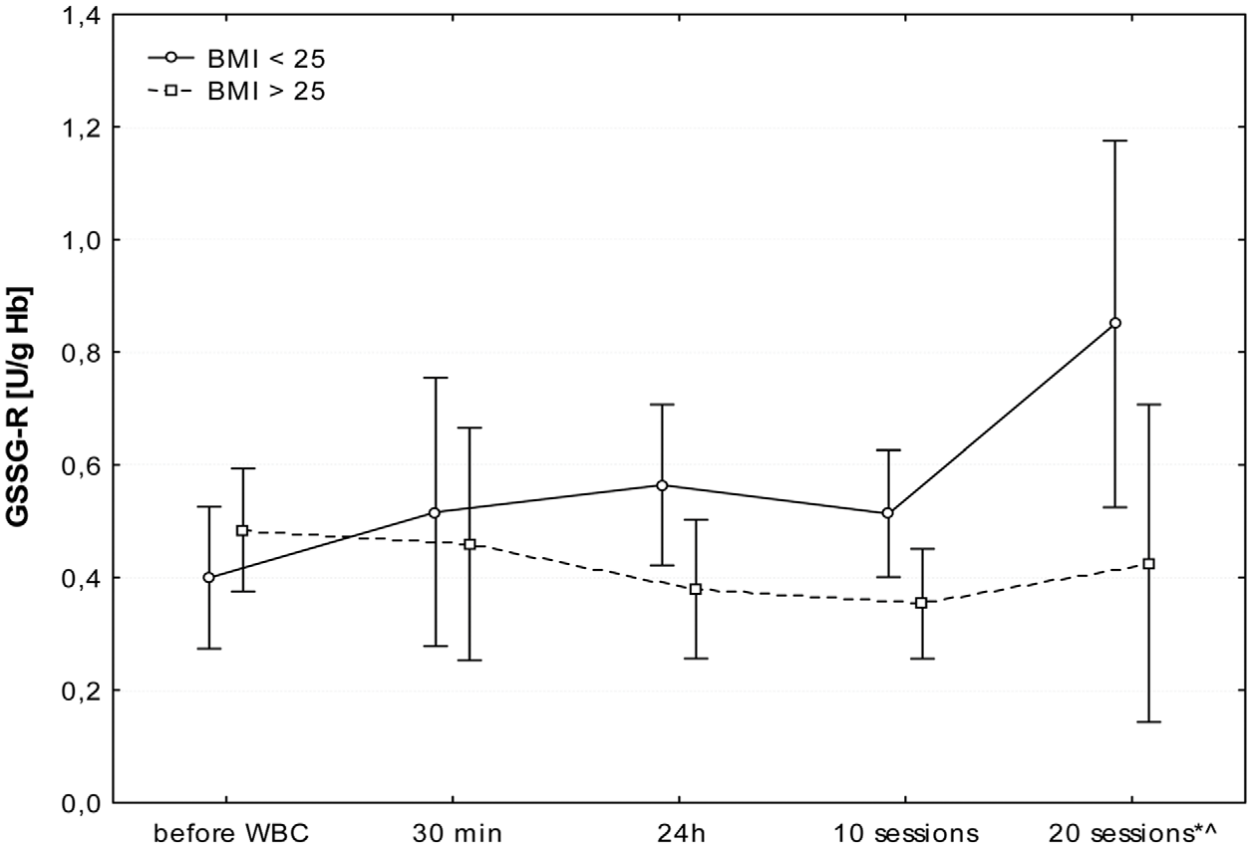
Changes in glutathione reductase (GSSG-R) before and during following sessions of WBC. Legend Figure 6: Median and 5%–95%; *p≤0.05 statistically significant difference vs. before WBC in subjects with BMI<25; ∧ p≤0.05 statistically significant difference BMI<25 vs. BMI>25; before WBC - A: before the first cryostimulation, after overnight fasting; 30 min - B: 30 min after the first cryostimulation; 24 h – C: 24 hours after the first cryostimulation, after overnight fasting; 10 session – F: 24 hours after the10^th^ cryostimulation, after overnight fasting; 20 session – I: 24 hours after the 20^th^ cryostimulation, after overnight fasting.

The first WBC session resulted in a significant increase in plasma Iso-P level after 24 h (B; p≤0.05). Further daily sessions sustained the elevated isoprostane, however at the 20^th^ stimulation, we observed a significant decrease in 8-isoprostane 24 h after stimulation (I; p≤0.05), in comparison to the level in the morning before 20^th^ stimulation (G). Figures 1, 2, 3, 4, 5, and 6 show changes in antioxidant enzyme activity in erythrocytes during the WBC and the level of reduced and oxidised glutathione. The activity of individual enzymes depended on exposure time. A series of 10 treatments resulted in no significant changes in the activity of superoxide dismutase, peroxidase or glutathione reductase, although in the case of SOD and GPx there was a downward trend. After 10 sessions there was a marked increase in catalase activity (p≤0.05), and level of glutathione, both reduced (43%) and in oxidised form (42%) at p≤0.05, without any accompanying changes in the ratios GSH:GSSG and SOD:CAT. After continuing WBC for 20 sessions, catalase activity returned to baseline, but the activity of superoxide dismutase significantly increased (43%), which entailed a significant increase in the SOD:CAT ratio. The continuing downward trend in glutathione peroxidase activity resulted in its significant reduction compared to the baseline.

The increase in concentrations of both forms of glutathione was only temporary and after 20 treatments the levels decreased significantly below baseline.

There was a very interesting, yet only one, relationship with respect to glutathione reductase. From the first session there was a difference in enzyme activity in people with a BMI<25 compared to those with a BMI>25, a difference which only gained in significance after a series of 20 treatments. In patients with BMI<25 there was a marked increase in the activity of this enzyme, while such changes did not occur in slightly overweight participants.

## Discussion

Short-term intensive cold exposure is used by physicians in physical therapy and sports medicine mainly to induce analgesic, anti-inflammatory and anti-swelling effects, and to reduce muscle tension. Very often, however, WBC is also used by healthy persons with the purpose of building up resistance to a natural thermal stimulus and the resultant increased resistance to diseases. Nowadays, exposure to the stimulating effects of environmental factors is increasingly rare due to the quality and comfort of modern life, and new technologies used in thermoactive clothing or sporting equipment. This increased protection is believed to increase the susceptibility to degenerative diseases, infectious diseases or “liabilities” of the central nervous system [21], hence the search for ways of preventing diseases and enhancing immunity based largely on the efficiency of the enzymatic and non-enzymatic components of the antioxidant defense system. Changes in metabolic activity and oxygen consumption during cold exposure may induce changes in reactive oxygen species production. It has been estimated that even in the conditions of systemic homeostasis 4%–5% of oxygen consumed during respiration is not completely reduced, instead forming free radicals [22], [23]. Reperfusion, associated with the two-stage character of the circulatory reaction of the body to cryostimulation, shivering thermogenesis and the activation of fat tissue metabolism, may lead to increased production of reactive oxygen species (ROS). Flavahan [24] reported that the generation of reactive oxygen species from mitochondria in vascular smooth muscle may mediate the vasoconstriction induced by local cooling. It is suggested that increased ROS activates RhoA/Rho kinase activation and consequent mobilisation of α2 –adrenoreceptors to the cell membranes where they can be activated by catecholamines from sympathetic neurons [25], [26], [27]. Apart from WBC, winter swimming in ice-cold water also leads to oxidative stress. Winter swimmers have higher baseline levels of reducing enzymes in erythrocytes (SOD, CAT and GPx) when compared with control subjects [21], [28], [29]. Regular winter swimmers have an increased resting number of white blood cells, monocytes and plasma interleukin-6 [30], [31]. However, conclusions from studies on the effects of winter swimming cannot be automatically compared with research on WBC. Immersion in cold water differs from exposure to cryogenic temperatures in the air, for example in the fact that in the aquatic environment an additional factor of increased physical activity and hydrostatic pressure exists. Tolerance of cold in the cryogenic chamber with very low humidity is higher than during immersion in cold water, which may have a temperature of about 4°C during the winter season.

There are not many reports on the impact of WBC on the immune system and peroxidant and antioxidant status without accompanying physical exercise and on clinically healthy individuals. In our previous studies, we have shown changes in the peroxidant and antioxidant status and the activity of SOD, CAT, GSH, T-glutathione transferase and GPx in response to a single WBC treatment, increased white blood cell counts, and levels of IL-6 in healthy participants undergoing a series of 10 daily WBC sessions [11], [12], [13]. In a rare similar study, Miller et al. [7] showed an increase in total antioxidant status, SOD activity and uric acid level in plasma in multiple sclerosis patients submitted for 10 sessions of cryostimulation. Under conditions of peroxidant-antioxidant homeostasis, the produced ROS are neutralised by enzymatic and non-enzymatic antioxidants, thus preventing lipid peroxidation, DNA damage and protein degradation [22]. The first line of defense, specifically against the formation of hydroxyl radicals, involves the activity of superoxide dismutase, catalase and glutathione peroxidase. As the result of a two-step dysmutation of the superoxide anion (⋅O_2_^−^) with the participation of SOD, hydrogen peroxide is produced (H_2_O_2_), which may be neutralised in the reaction of disproportionation with CAT and reduction with GPx [32], [33]. Although CAT and GPx are both able to destroy H_2_O_2_, GPx has much higher affinity for hydrogen peroxide than CAT, and mainly degrade this reactive oxygen species under normal conditions. Measurements of isoprostanes in plasma during the following days of cryostimulation provided a quantitative index of oxidant stress. Isoprostanes are prostaglandin (PG) –like substances that are produced in vivo independently of cyclooxygenase (COX; EC 1.14.99.1) enzymes, primarily by free radical- induced peroxidation of arachidonic acid. Isoprostanes are initially formed esterified on phospholipids and then released in free form by phospholipase(s) [34], [35]. Isoprostanes are not only the indicator of increased free radical reactions. They also play a role of mediators of oxidant damage both in physiological and pathophysiological processes. Isoprostanes have anti-inflammatory properties [36], as they counteract the adhesion of monocytes to epithelial cells of blood vessels in vitro [37].

On the other hand, their free radical formation mechanism in membrane phospholipds is a cause of changes in the liquidity and integrity of cellular membranes [38]. Even a single cryostimulation was a stress-inducing factor that increased the concentration when checked 24 hours later. The subsequent stimulations sustained the high 8-Iso-P concentration. Mean isoprostane concentrations in the plasma of healthy humans range from 35±6 to 119±22 pg/ml [39]. In this paper, these values were distinctly higher, especially in response to cryostimulation. It is interesting that after the 20^th^ treatment, we observed a slightly different reaction than after the 10^th^. The day after the 20th cryostimulation (I) we observed a significant decrease in 8-Iso-P compared to values before this 20^th^ stimulation (G) (Tab. 2). It may be supposed that prolonged daily exposure to cold resulted in improved defense mechanisms and antioxidant parameters – greater resistance to cold and oxidant damage resulting from other factors, but only after long enough period of time.

This is consistent with our earlier reports on WBC as a stress-inducing factor that activates favourable adaptation mechanisms and increases the resistance of the human body. Importantly, in our earlier papers, we observed no significant increase in total oxidative status in plasma after 10 daily cryostimulations compared to the initial level, although we found some changes in total antioxidant capacity in plasma [13]. Similary, Miller et al. [16] showed no increase in TBARS in plasma after 10 sessions of WBC treatment in healthy men. It could suggest that both TOS and TBARS are not good markers to assess oxidative stress status in response to cold, as they are not a specific enough product of lipid peroxidation and 8-Iso-P could be a more sensitive and appropriate biomarker to assess stress levels in response to cold stimulation, especially as they are considered a sensitive marker of lipid peroxidation derived from the oxidation of adipose tissue. It seems worth noting that in our previous studies we observed that whole body exposure to cryogenic temperatures leads to changes in lipid metabolism, especially with an increasing number of WBC sessions [14].

It could be postulated, that in our study, the increased free radical reactions after cryogenic temperature exposure lead to increase in 8-Iso-P, induce a response - changes in the antioxidant capacity, a longer series (of 20 WBC) sessions changed the capacity of the antioxidant system by adaptation changes.

The elevated levels of GSH and GSSG after 10 treatments were accompanied by an increase in CAT activity and a slight decrease in SOD/CAT ratio. The absence of increased activity of glutathione reductase, glutathione peroxidase and superoxide dismutase, suggests that the exposure time was too short to induce favourable adaptive changes in the antioxidant system, and also because the situation changed after a series of 20 sessions. The longer series of WBC sessions resulted in an increase in SOD activity, SOD/CAT ration and the activity of GSSG-R, albeit only in those with a BMI<25, but it is worth to notice that the large SD value was noticed and the future study are necessary to confirm those dependence. Interestingly, there was a decrease in GSH and GSSG (but without changes in the GSH:GSSG ratio) and in glutathione peroxidase. GPx plays a very significant role in red blood cells, protecting hemoglobin against the damaging effect of hydrogen peroxide, both during peroxidant-antioxidant homeostasis and increased free radical reactions. GPx seems to be most clearly influenced by life-style and environmental factors, e.g. smoking habits [40]. Increasing activity on the pathway of *de novo* synthesis of an enzyme is energy-demanding and slow, GPx is reserved as the last means to cope with oxidative stress. SOD activities appear sufficiently high and relatively uniform across tissues, suggesting that the removal of superoxide anion may not be a rate-limiting step. In comparison, GPx destroys the end products of the ROS generation pathway and its activity is relatively low [41]. Increased glutathione reductase activity in subjects with normal BMI and absence of this reaction in people with elevated BMI may suggest the influence of a factor released from adipose tissue that inhibits enzyme activity or synthesis. It is worth noting that these differences become clear only after the continuation of 20 WBC treatments. Similarly, distinct changes in lipid profile after exposure to cryogenic temperatures occurred only after 20 treatments [14].

Moreover, further research that would take into account detailed body composition could help verify whether varied R-GSSG activity in the participants depended on the cooling rate of the body and on the fat and lean mass of the individuals.

We also observed a considerable change in plasma non-enzymatic antioxidants, such as uric acid, a well-established scavenger of reactive oxygen and nitrogen species such as hydroxyl radical and peroxynitrite [42], [43], [44], [45]. The concentration of uric acid decreased after 10 WBC sessions but then significantly increased after the series of 20 WBC sessions, with a simultaneous decrease in plasma albumin and ceruloplasmin. It seems that the oxidant species produced during the first stage of WBC treatment (confirmed by a significant increase in 8-Iso-P) induces uric acid consumption. The results of the presented studies indicate a significant role of uric acid as a principal antioxidant molecule in the human body.

Our results are inconsistent with the findings of Siems et al. [29] who examined healthy subjects who swam regularly in ice-cold water during winter (winter swimming). A drastic decrease in plasma uric acid concentrations during and following exposure to the cold stimulus was explained as the consumption of uric acid after the formation of oxygen radicals. However, during winter swimming, cold is accompanied by increased physical activity, during which mechanisms of thermoregulation and heat loss differ significantly from a 3 minute long exposure to a temperature of −130°C in dry air. Besides, the season of winter swimming was longer than the general time of the WBC series in our experiment.

In our study, the prolonged exposure of 20 WBC sessions led to a reduction in the level of albumin in blood serum with the simultaneous increase in total protein as early as after 10 treatments, without changes in the blood count that would indicate haemoconcentration. Albumin may be a target of the hydroxyl radical, which causes impairment and a resultant proteolytic degradation. As is known, one of the factors affecting protein synthesis in the liver is IL-6 (e.g. during the acute phase reaction) which reduces albumin synthesis and increases the synthesis of acute phase proteins - including α1-globulins [46], [47]. In our previous studies there was an increase in circulating levels of IL-6 [13], [15] in healthy individuals undergoing cryostimulation there was a reduction in albumin concentration, increased concentrations of α1-globulins and lower levels of ceruloplasmin [48]. Therefore it is very likely that the increase in total protein occurring in the consecutive days of the WBC could be linked to the production of globulin (as a acute phase reactants) or even immunoglobulins. Albumin forms complexes with copper and iron, preventing their participation in the Fenton reaction that increases the production of hydroxyl radicals – a factor that impairs tissues [49]. Glucose and albumin bind the hydroxyl radical and ceruloplasmin inhibits lipid peroxidation dependent on copper and iron. Albumin and probably also 2-macroglobulin are proteins that transport copper in plasma [50]. This element is essential for the antioxidant activity of enzymes such as SOD or lysine oxidase [51]. These proteins provide copper to the sites of SOD synthesis which is induced by interleukin 1 (IL-1) or tumor necrosis factor α (TNF-α) [52]. The consumption of copper by antioxidant enzyme (SOD) synthesis and the decreased concentration of albumin (main transporter of copper in serum) may alleviate the synthesis of ceruloplasmin.

In therapeutic practice, the most common application of cryostimulation is a series of 10 WBC sessions, without providing any scientific justification. In the light of our findings and earlier reports, it seems that favourable adaptive changes, for example in the activity of antioxidant enzymes, lipid profile, appear only after a longer series of WBC (here 20 sessions). In order to the determine the most advantageous procedure for cryostimulation, it is necessary to continue research on the optimum number of sessions in a series and the effects of multiple application of cryostimulation during one day (2 or 3 per day) that is often used by athletes during recovery.

It is interesting that changes in the activity of glutathione reductase depend on the BMI of participants. It seems that further studies are necessary on the effect of cryostimulation on people of differing body content and mass. Our investigation indices that measurement of 8-isoprostanes could be more reliable approach to assess oxidative stress status in vivo, during cold exposure than evaluation of the other biomarkers.
